# The Evolution of JCHIMP

**DOI:** 10.3402/jchimp.v1i2.7292

**Published:** 2011-07-18

**Authors:** Robert P. Ferguson

In the last issue, which was the first issue of JCHIMP, I discussed the origins and background of the journal (1).

The journal was conceived in late summer 2010 and emerged with the first issue in May 2011. At the outset we were not certain of our direction beyond the fact that we wanted to be a clinically oriented, general internal medicine journal supporting the interests of community teaching hospitals and the trainees and faculty who work in them. It can be difficult to conduct scholarly work in the community setting in comparison to many university-based programs because of institutional culture, emphasis on clinical volumes, and lack of resources. Nevertheless, good scholarly work has been evident in venues like the American College of Physicians Associates Meeting each year where residents and students present clinical vignettes and other scholarly work of high quality. Too often, the scholarly effort ends there; JCHIMP provides further options. Contracting with Co-Action Publishing in Stockholm has had a major positive impact. Our 15 member editorial board and over 110 reviewers are a testimony to broad support and interest – even at this early stage. In our first issue, David Solomon, champion of open access medical journals, pointed out the glowing future of electronic open access journals as the numbers are increasing and the quality improving (2). We believe that our journal, JCHIMP, will fill a much needed niche of internal medicine open access journals that focus on community programs and the people who work and study there.

Our objective is to publish four to six times a year. We prefer shorter, succinct papers. All will be clinically oriented and in most cases will have been accomplished at the community hospital site. The review process will always include a general internist because we want our generalist readership to appreciate the research.

This second issue is one of the theme issues for the first year and deals primarily with patient safety by a number of experts in the hospital setting. Ethan Fried (3) has written an overview of the issues of patient safety in community hospitals and points out that the culture of patient safety is widespread but that ‘not all boats are raised equally in the tide’. Three of the papers were presented at the Association of Program Directors (APDIM) Chiefs and Chairs Forum in San Antonio, Texas in October 2010. They are: hypoglycemia in the hospital by Mansur Shomali (4), urinary tract infections and hand washing by Eskandar Yazaji (5), and antibiotic stewardship by Trevor Van Shooneveld (6). Additionally, there is an EKG column by Marc Mugmon (7) that demonstrates that paroxysmal rhythms are usually not physiologic and how they can be identified. Ramesh Khurana (8) has an original research study on standardizing measurements related to the Valsalva Maneuver.

We will be publishing observational studies and perspective pieces. We are looking for epidemiologic reports and will be doing case reports that are good examples of practice-based learning. We will also be doing brief reports and letters to the editor. We hope to publish a solid contemporary peer review journal that is very readable. We anticipate no longer than a few months between the submission of an article and its publication. Obviously, this depends somewhat on the cycle of the calendar. Our first issue had a good example of the value of short publication cycles. A case of leptospirosis presenting to a hospital emergency room in November 2010 was published in our first issue in May 2011 (9). A potential rodent-centered urban zoonosis is news.

We will use at least three reviewers for each manuscript, two for invited manuscripts. We believe that blinding reviewers is crucial. For many years, I have participated in abstract judging competitions of research and clinical vignettes and concluded that there is a great deal of uneven subjectivity. We will be submitting for indexing to PubMed Central and other indices through Co-Action Publishing once our volume of published articles reaches sufficient levels.

We are hoping that this journal will be a platform for community hospital scholarly work nationally. We originated in Baltimore, Maryland but we are encouraging submission of research and other scholarly work from around the country. We plan to work closely with our publisher, Co-Action, on distribution to develop a wide audience – perhaps even an international one.

We feel we are off to a great start. We have had a variety of manuscripts that have been excellent and interesting and that word is getting out regarding readers and reviewers. We have received tremendous cooperation from dozens of individuals and are in a very steep learning curve as many of the participants involved in this review effort are doing it with little previous experience. We believe that this initiative – incorporating many faculty into the peer review process – effectively serves as a faculty development project unto itself, allowing some individuals to discover talents, skills, and interests of which they were not previously aware.

We feel that we will inevitably get more efficient with our operation. We do not believe that we have scratched the surface of the technological advances provided by this medium. We hope to formally link up with the 100 institutions that are part of the Community Hospital Education and Research Network (CHERN, www.CHERNINFO.com). With similar goals, CHERN and JCHIMP have the potential for program development and synergy.

*Robert P. Ferguson, MD* Editor

